# Transcriptome analysis reveals the impact of NETs activation on airway epithelial cell EMT and inflammation in bronchiolitis obliterans

**DOI:** 10.1038/s41598-023-45617-y

**Published:** 2023-11-06

**Authors:** Zhongji Wu, Xiaowen Chen, Shangzhi Wu, Zhenwei Liu, Hongwei Li, Kailin Mai, Yinghui Peng, Haidi Zhang, Xiaodie Zhang, Zhaocong Zheng, Zian Fu, Dehui Chen

**Affiliations:** 1 The First Affiliated Hospital of Guangzhou Medical University, Guangzhou, 510000 People’s Republic of China; 2 Guangzhou Medical University, Guangzhou, 510000 People’s Republic of China

**Keywords:** Cell signalling, Respiratory tract diseases

## Abstract

Bronchiolitis obliterans (BO) is a chronic airway disease that was often indicated by the pathological presentation of narrowed and irreversible airways. However, the molecular mechanisms of BO pathogenesis remain unknown. Although neutrophil extracellular traps (NETs) can contribute to inflammatory disorders, their involvement in BO is unclear. This study aims to identify potential signaling pathways in BO by exploring the correlations between NETs and BO. GSE52761 and GSE137169 datasets were downloaded from gene expression omnibus (GEO) database. A series of bioinformatics analyses such as differential expression analysis, gene ontology (GO), Kyoto encyclopedia of genes and genomes (KEGG), and gene set enrichment analysis (GSEA) were performed on GSE52761 and GSE137169 datasets to identify BO potential signaling pathways. Two different types of BO mouse models were constructed to verify NETs involvements in BO. Additional experiments and bioinformatics analysis using human small airway epithelial cells (SAECs) were also performed to further elucidate differential genes enrichment with their respective signaling pathways in BO. Our study identified 115 differentially expressed genes (DEGs) that were found up-regulated in BO. Pathway enrichment analysis revealed that these genes were primarily involved in inflammatory signaling processes. Besides, we found that neutrophil extracellular traps (NETs) were formed and activated during BO. Our western blot analysis on lung tissue from BO mice further confirmed NETs activation in BO, where neutrophil elastase (NE) and myeloperoxidase (MPO) expression were found significantly elevated. Transcriptomic and bioinformatics analysis of NETs treated-SAECs also revealed that NETs-DEGs were primarily associated through inflammatory and epithelial-to-mesenchymal transition (EMT) -related pathways. Our study provides novel clues towards the understanding of BO pathogenesis, in which NETs contribute to BO pathogenesis through the activation of inflammatory and EMT associated pathways. The completion of our study will provide the basis for potential novel therapeutic targets in BO treatment.

## Introduction

Bronchiolitis obliterans (BO) is a chronic airway disease, often represented through pathological changes in the narrowing and obstructive airways that were found rare and irreversible. The BO patient usually has symptoms such as recurrent cough, persistent wheezing and sputum production, decreased exercise tolerance, shortness of breath, or other types of breathing difficulties^1^. BO is the cause for concerns primarily due to the patients’ reduced long-term survival after lung transplantation^2, 3^. Besides, BO can present itself as complication following adenoviral pneumonia in children, hence there is an urgent need to further understand BO pathogenesis and progression^4^. However, despite the efforts put into BO research, the exact underlying mechanisms that modulated BO pathogenesis remain obscure. Consequently, the effective treatments available for BO are scarce and severely lacking. Therefore, in order to formulate effective therapeutic strategies, a further in-depth understanding of BO pathogenesis is deemed critical.

Our approach focuses on the use of bioinformatics analyses and were coupled with the use of cellular and animal models. Specifically, bioinformatics analyses derived from next-generation sequencing technologies can offer researchers a more comprehensive understanding of the identification of molecular mechanisms crucial for diseases pathogenesis and progression. For example, research in BO recently had suggested the involvement of innate immune reactions in BO pathogenesis, in which the combinational analysis of transcriptomic sequencing and bioinformatics had provided the means to better identify the gene and its respective signaling pathways in BO pathogenesis^5, 6^. Moreover, by analyzing and comparing existing BO-related transcriptomic data from various studies, researchers may gain opportunities to discover novel target, that was previously neglected in the study of BO. Hence, two BO-related transcriptomic datasets were selected and downloaded from the gene expression omnibus (GEO) database for this study. Specifically, these datasets were used to examine the potential involvements of the neutrophil extracellular traps (NETs) formation signaling pathways in BO pathogenesis. We further confirmed the involvement of NETs in BO pathogenesis by evaluating data obtained from BO mouse models that were constructed using two publicly well-recognized approaches, which are 2,3 butanedione treatment and nitric acid treatment. Consistent with our bioinformatics analysis, crucial indicators of NETs molecules such as neutrophil elastase (NE) and myeloperoxidase (MPO) were found elevated in the lung tissues of BO mice. Altogether, our efforts had revealed the potential links between NETs regulation and the activation of inflammatory and epithelial-mesenchymal transition (EMT)-related pathways, that are vital for pathogenesis and progression of BO.

## Materials and methods

### Animals

Male SPF C57 mice (6–8 weeks old and weighing 18–22 g) were obtained from the Southern Medical University Animal Center (Guangzhou, China) and housed in appropriate cages with water and routine diet. The protocols were approved by the institutional ethics committee (ethics number: 20230018). This animal study procedures were performed according to ARRIVE guideline. All the methods described in this study were performed in accordance with the relevant guidelines and regulations of the Institution.

### Nitric acid-induced bronchiolitis obliterans model

Mouse models of nitric acid-induced bronchiolitis obliterans were established according to established procedures in the field^7^. Briefly, mice were anaesthetized with isoflurane (5% isoflurane for induction, 2.5% isoflurane for maintenance) and immobilized on a mouse tracheal intubation table, and a 24G intravenous needle was inserted through the mouth into the trachea to drip 1 μL/g of 0.5% nitric acid. After a single drip of nitric acid, BO-affected mice were established 21 days after instillation. To obtain lung samples, mice were euthanized by an overdose of 5% isoflurane and cervical dislocation. To remove blood from the lungs, they were flushed with 3 mL of PBS via the right ventricle. Lung samples were collected and snap frozen in liquid nitrogen until further processing. Adequate amounts of lung samples from each mouse were used for protein quantification. In addition, fresh lung samples were soaked in 4% paraformaldehyde for biochemical analyses such as H&E and Masson’s staining.

### 2,3-butanedione-induced bronchiolitis obliterans model

The application of 2,3-butanedione to generate mouse models of bronchiolitis obliterans was conducted in accordance with established methodologies and protocols detailed in earlier scientific publications within this field of study^8^. Anesthesia, tracheal dropping and sampling were performed as above. 2,3-butanedione was titrated to a concentration of 30% for titration of 0.333 μL/g. Animals were euthanized by cervical dislocation after isoflurane overdose 14 days after 2,3-butanedione instillation.

### Quantify the degree of occlusion observed in the bronchioles

In our study, we utilized the percentage of luminal occlusion area as a quantitative measure to assess the degree of bronchiolar obstruction. The specific method involved selecting three non-overlapping fields of view at 4 × magnification on HE-stained lung tissue slides. Within each field of view, we calculated the percentage of occluded area in relation to the total luminal area of the bronchioles. The average occlusion percentage from these three fields of view was then taken as the final quantification of the extent of obstruction. This approach enables us to evaluate and quantify the observed bronchial occlusion.

### BO gene expression profile dataset selection

We searched the GEO database (https://www.ncbi.nlm.nih.gov/geo/) for gene expression profile data of BO and screened the identified datasets according to the inclusion and exclusion criteria. The inclusion criteria were as follows: (1) containing three or more pairs of samples from the BO group and the normal group, (2) the dataset type is expression profiling by array or RNA profiling by array, (3) the sample type is lung or bronchial. Finally, two datasets related to BO in lung or bronchial tissue transcriptome data were retrieved from the GEO database. The information on two datasets is shown in Table 1.

**Table 1:** Data resource.

ID	Status (N:P)	Organism	Tissue	Platform
GSE52761	6:3	*Rattus* *norvegicus*	Bronchial	GPL1355
GSE137169	3:3	Mouse	Lung	GPL21810

### Data processing and differentially expressed genes identification

Raw microarray data for the BO datasets were downloaded from the GEO database. We normalised the data using the R package limma^9^ in R software 4.2.3 and converted to log2 values for further analysis. We used the R package limma to obtain DEGs between the BO and control groups from the GSE52761 and GSE137169 datasets. Genes with p < 0.05 and |log2 fold change (FC) |≥ 1.5 were considered as differentially expressed genes (DEGs) in the respective databases. The overlapping DEGs between the two datasets were considered the final DEGs.The volcano maps and Venn diagrams were generated using Sangerbox 3.0 (http://sangerbox.com/)^10^.

### Enrichment pathway analysis of up-regulated DEGs and down-regulated DEGs

Gene ontology (GO) analysis is an effective method for annotating genes and identifying characteristic biological attributes, including biological processes (BP), molecular functions (MF) and cellular components (CC)^11^. The Kyoto encyclopedia of genes and genomes (KEGG) database provides a comprehensive collection of data on protein interaction networks and biointerpretation of genomic sequences^12^. In our study, GO and KEGG analysis of DEGs were completed by the ‘clusterProfiler’ package^13^. For all cases, we recorded the top 5 significant items in BP, CC and MF and the top 20 significant pathways in KEGG analysis.

### Gene set enrichment analyse of datasets

Gene Set Enrichment Analysis (GSEA) is a gene set-based functional pathway enrichment analysis method that calculates the enrichment fraction of gene sets in each functional pathway^14^. GSEA was performed using GSEABase, clusterProfiler and org.Mm.eg.db packages. The pathways with P value < 0.05 and enrichment scores > 0.45 were defined as the predictive up-regulated pathway from GSEA.

### Western blot analysis

Lung tissue samples were lysed in a buffer containing Tris–HCl, SDS, glycerol, and other reagents. The resulting lysate was then centrifuged and the protein concentration was determined using the BCA method. Equal amounts of protein were separated using SDS-PAGE and transferred to PVDF membranes. The membranes were blocked and incubated with primary antibodies against myeloperoxidase (MPO) (R&D: AF3667-SP; 0.5 µg/mL) and neutrophil elastase (NE) (CST: E8U3X; 1:1000), followed by a secondary antibody, and protein bands were detected using chemiluminescence. ImageJ software was used to calculate the ratio of MPO and NE to β-actin, and significance was determined by Student’s *t* test (P < 0.05) using GraphPad Prism software.

### Extraction of NETs from neutrophils

We utilized a commercially available human neutrophil isolation kit (Haoyang, Tianjin, China) to isolate neutrophils from freshly collected whole blood obtained from healthy blood donors. The isolation procedure was performed according to the manufacturer’s instructions. Subsequently, we employed BeyoMag™ Protein A + G Magnetic Beads (Beyotime, Shanghai, China) to isolate IgG from the plasma of bronchiolitis obliterans (BO) patients who were confirmed as ANCA-positive using ELISA detection method.The isolated neutrophils were then resuspended in RPMI 1640 medium at a density of 1.8 × 106 cells per milliliter and stimulated with ANCA-positive IgG at a concentration of 5 μg/mL. The stimulation was carried out at a temperature of 37 °C for a duration of 24 h.To separate and quantify neutrophil extracellular traps (NETs), the supernatant was removed, and the NETs layer at the bottom of the plate was collected using phosphate-buffered saline (PBS). The collected NETs were subsequently transferred to a centrifuge tube. Following centrifugation at a speed of 20×*g* for 5 min, a cell-free supernatant enriched in NETs was obtained.To quantify the amount of NETs, we employed the PicoGreen dsDNA analysis method (Invitrogen, USA) to measure the levels of free DNA. This part of the experiment has been approved by the ethical review of the First Affiliated Hospital of Guangzhou Medical University (ES-2023-011-01).

### Human airway epithelial cells treatment with NETs

Human small airway epithelial cells (SAECs) were purchased from Procell Life Science & Technology Company (Wuhan, China) and cultured in DMEM high glucose supplemented with 10% FBS, 1% penicillin–streptomycin solution, and 1% l-glutamine (all purchased from Procell, Wuhan, China) at 37 °C with 5% CO_2_. SAECs were seeded in 24-well plates to 90% confluency, washed once with PBS, and 20 μg/mL NETs or DMEM were added. The total volume in each well was kept equal by adding DMEM medium. After 24 h treatment, SAECs were harvested for RNA sequencing.

### RNA sequencing of SAECs

Total RNA was extracted from NETs-treated SAECs and untreated SAECs. 1–2 ug of total RNA was selected from each sample to construct RNA sequencing libraries. The constructed libraries were quality controlled by Agilent 2100, and the libraries were quantified by the qPCR method and sequenced on an Illumina NovaSeq 6000 sequencer. The raw sequencing files were imported into StringTie to calculate transcript abundance by comparing the results to known transcriptomes, and then the data were converted to fragments per million mapped reads per thousand bases of transcription (PFKM) by Ballgown calculation, and the genes with a PFKM mean > 0.5 were selected for further analysis. The sequencing data were uploaded to GEO database (https://www.ncbi.nlm.nih.gov/geo/query/acc.cgi?acc=GSE232805).

### Identification of NETs-related DEGs

After processing the data using the above approach, genes with p < 0.05 and |log2 fold change (FC) |≥ 1 were considered NETs-DEGs.

### Functional and pathway analysis of NETs-DEGs

GO and KEGG were used to analyse the pathways enriched by NETs-DEGs and sorted by P value to show the ranked top 5 results.

### Protein–protein interaction network analysis of NETs-DEGs

Protein–protein interaction (PPI) network analysis of NETs-DEGs was performed using the online STRING website (https://string-db.org/)^15^. Then, Cytoscape 3.8.0 (14) was used to visualize the PPI network. The screening condition for constructing the PPI network was a combined score > 0.4.

### Identifying the key module

Molecular complex detection (MCODE) is a plugin for Cytoscape to build important functional modules in PPI networks^16^. Parameters were set as Node score cutoff = 0.2, Degree cutoff = 2, K-core = 4, and Max. Depth = 100. The modules with SCORE > 10 were defined as the key modules of PPI.

### Enrichment pathway analysis of key module

KEGG was used to analyze the pathways enriched by the key module genes and sorted by P value to show the top 6 results.

### Disease ontology analysis of key module

Like GO, disease ontology (DO) is a formal ontology of diseases^17^. We performed enrichment analysis on the key module genes as mentioned above based on DO terms and sorted by P value to show the top 15 DO terms.

### Ethics approval and consent to participate

The study was approved by the Institutional Ethics Committee of the First Affiliated Hospital of Guangzhou Medical University (protocol code: 20230018 and ES-2023-011-01). Mouse husbandry followed ARRIVE guidelines (http://www.nc3rs.org.uk/arrive-guidelines).

## Results

### Identification of differentially expressed genes in BO

**Figure 1:**
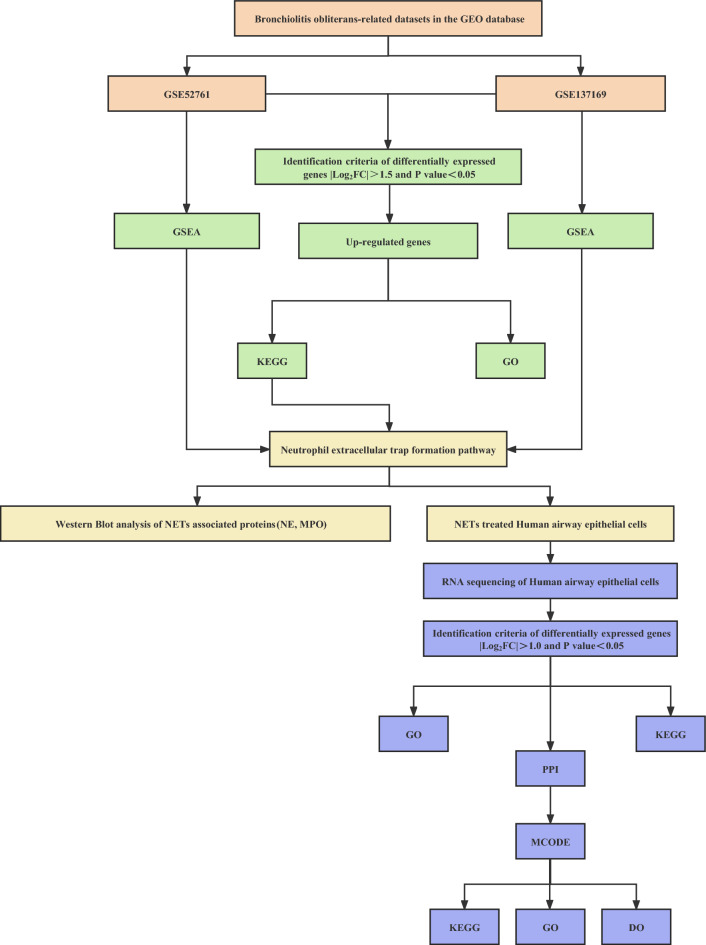
The flowchart of the analysis process.

**Figure 2:**
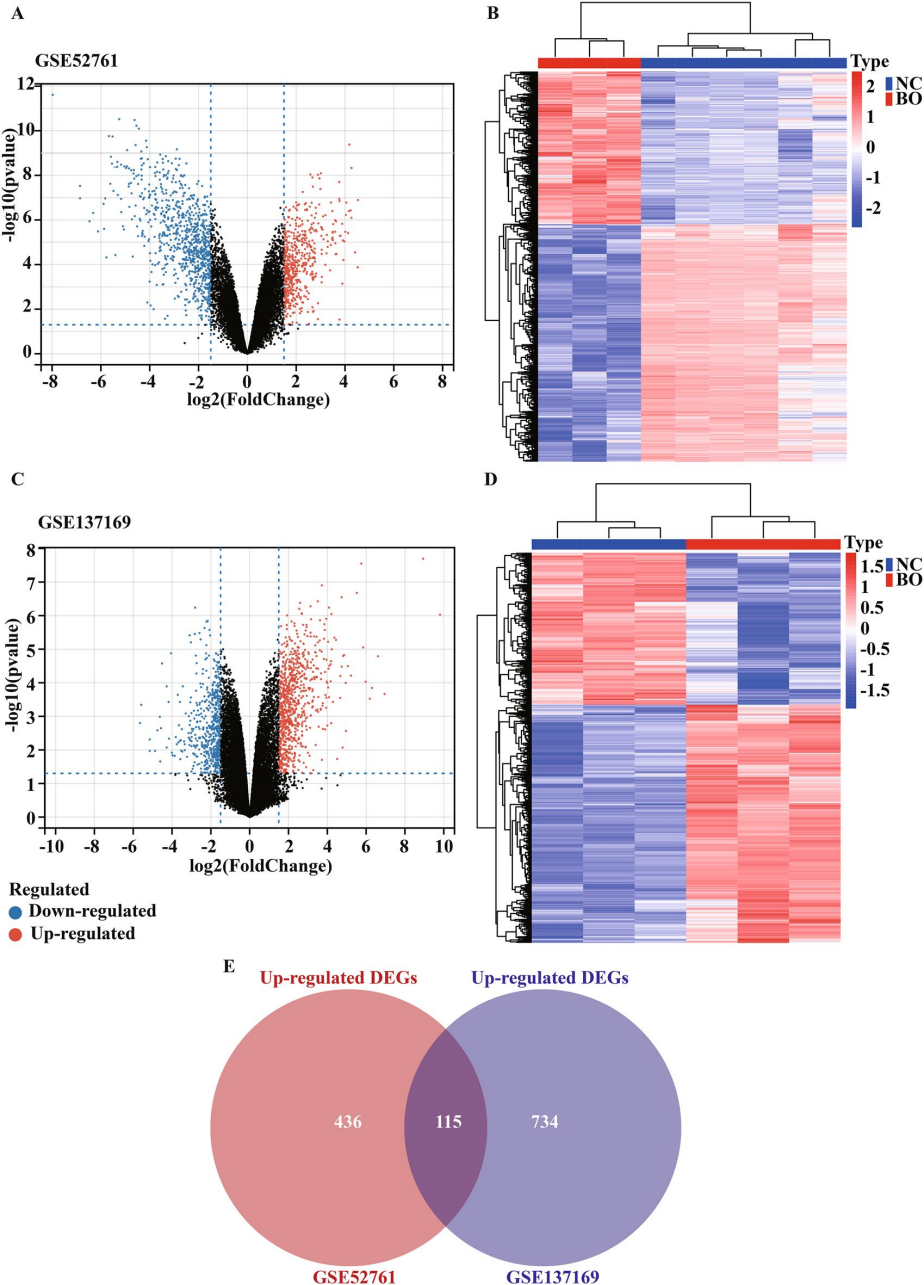
Identification of DEGs between the normal group and BO group in the analysis datasets. (**A**,**B**) The volcano plots (**A**) and heat mapping (**B**) of DEGs in GSE52761. (**C**,**D**) The volcano plots (**C**) and heat mapping (**D**) of DEGs in GSE137169. (**E**) Venn diagrams showed the overlap of numbers of up-regulated DEGs between GSE52761 and GSE137169.

We performed a systematic review of GEO datasets for BO. A schematic of the screening strategy to identify such compounds is shown in Fig. 1. We selected two acceptable datasets, GSE52761 and GSE137169, which contained at least 3 pairs of normal or BO lung expression profiles by RNA array (Table 1). We identified and visualiszd the DEGs in related datasets by volcano mapping and heat mapping in the respective databases (Fig. 2A–D). We obtained a total of 115 up-regulated DEGs and 49 down-rengulated DEGs (Fig. 2E, Supplementary Fig. 1A and Table 1).

### GO and KEGG results of DEGs

**Figure 3:**
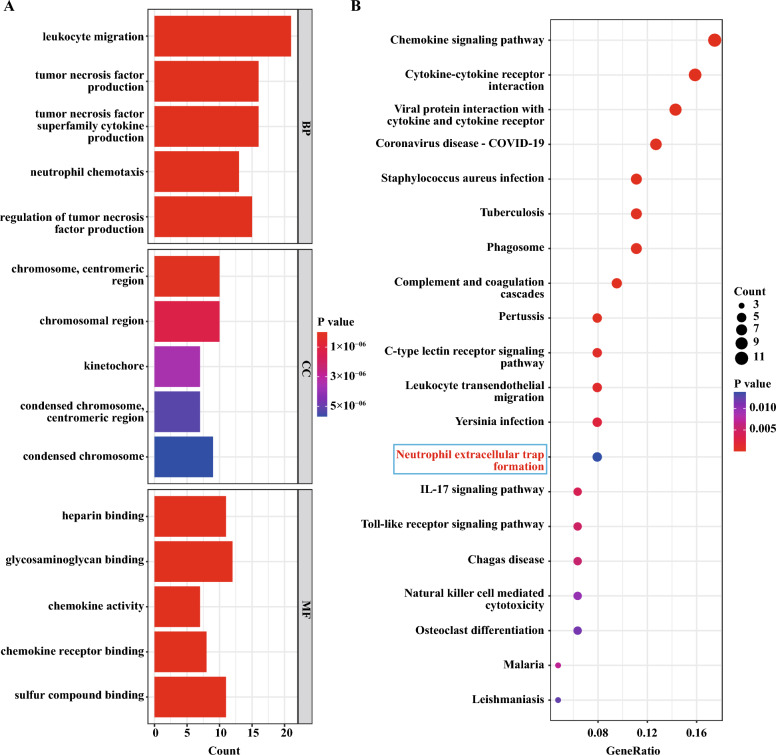
GO and KEGG enrichment results of up-regulated DEGs. (**A**) The bar graph showed the top 5 significant items in the BP, CC, and MF fractions based on the P values in the GO analysis. (**B**) The bubble plot showed the top 20 significant pathways in the KEGG analysis.

To better understand the function of up-regulated DEGs, we subjected the 115 up-regulated DEGs to GO and KEGG analyses. According to the results of GO analysis results, the changes in BP of up-regulated DEGs were significantly enriched in the ‘leukocyte migration’, ‘tumor necrosis factor production’, ‘tumor necrosis factor superfamily cytokine production’, ‘neutrophil chemotaxis’ and ‘regulation of tumor necrosis factor production’; CC of up-regulated DEGs were enriched in ‘chromosome, centromeric region’, ‘chromosomal region’, ‘kinetochore’, ‘condensed chromosome, centromeric region’ and ‘condensed chromosome’; and the changes in MF were enriched in ‘heparin binding’, ‘glycosaminoglycan binding’, ‘chemokine activity’, ‘chemokine receptor binding’ and ‘sulfur compound binding’ (Fig. 3A). KEGG pathway analysis revealed that the pathways enriched by up-regulated DEGs include ‘Chemokine signaling pathway’, ‘Viral protein interaction with cytokine and cytokine receptor’, ‘Coronavirus disease—COVID-19’, ‘Staphylococcus aureus infection’, ‘Tuberculosis’, ‘Phagosome’, ‘Complement and coagulation cascades’, ‘Pertussis’, ‘C-type lectin receptor signaling pathway’, ‘Leukocyte transendothelial migration’, ‘Yersinia infection’, ‘Neutrophil extracellular trap formation’, ‘IL-17 signaling pathway’, ‘Toll-like receptor signaling pathway’, ‘Chagas disease’, ‘Natural killer cell mediated cytotoxicity’, ‘Osteoclast differentiation’, ‘Malaria’, and ‘Leishmaniasis’ (Fig. 3B). Supplementary Fig. 1B and C demonstrated the results of a similar analysis conducted for the down-regulated DEGs.

### Gene set enrichment analysis results of the two BO datasets

**Figure 4:**
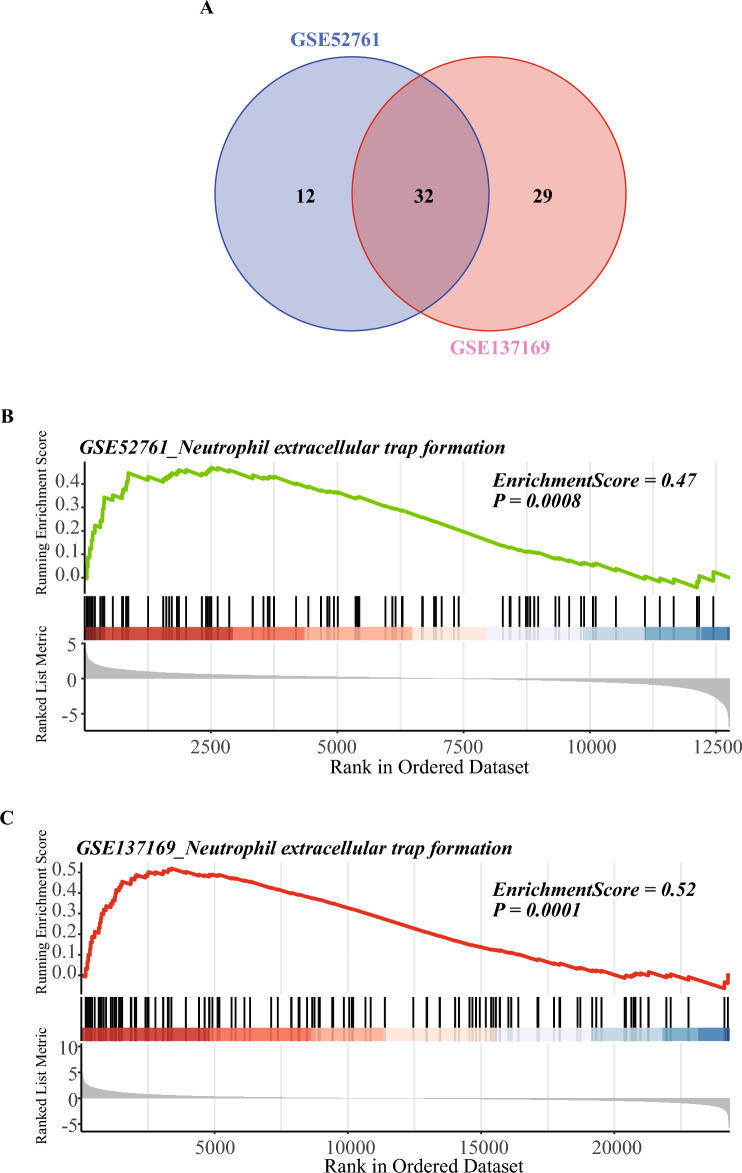
GSEA results of GSE52761 and GSE137169. (**A**) Venn diagrams showed the overlaps of predictive up-regulated pathway between GSEA results of GSE52761 and GSEA results of GSE137169. The predictive up-regulated pathway was identified with P value < 0.05 and enrichment scores > 0.45. (**B**) The GSE52761 dataset exhibits significant enrichment in genes related to the Neutrophil extracellular trap formation (P value = 0.0008, ES = 0.47). (**C**) The GSE137169 dataset exhibits significant enrichment in genes related to the Neutrophil extracellular trap formation (P value = 0.0001, ES = 0.52).

GSEA predicted 44 predicted up-regulated pathways from the GSE52761 dataset and 61 predicted up-regulated pathways the from GSE137169 dataset, and their intersecting Venn diagrams showed a total of 32 predicted BO up-regulated pathways (Fig. 4A and Supplementary Table 2), including the Neutrophil extracellular trap formation (Fig. 4B,C).

### Myeloperoxidase and neutrophil elastase were upregulated in the 2,3-butanedione-induced BO model and nitric acid-induced BO model

**Figure 5:**
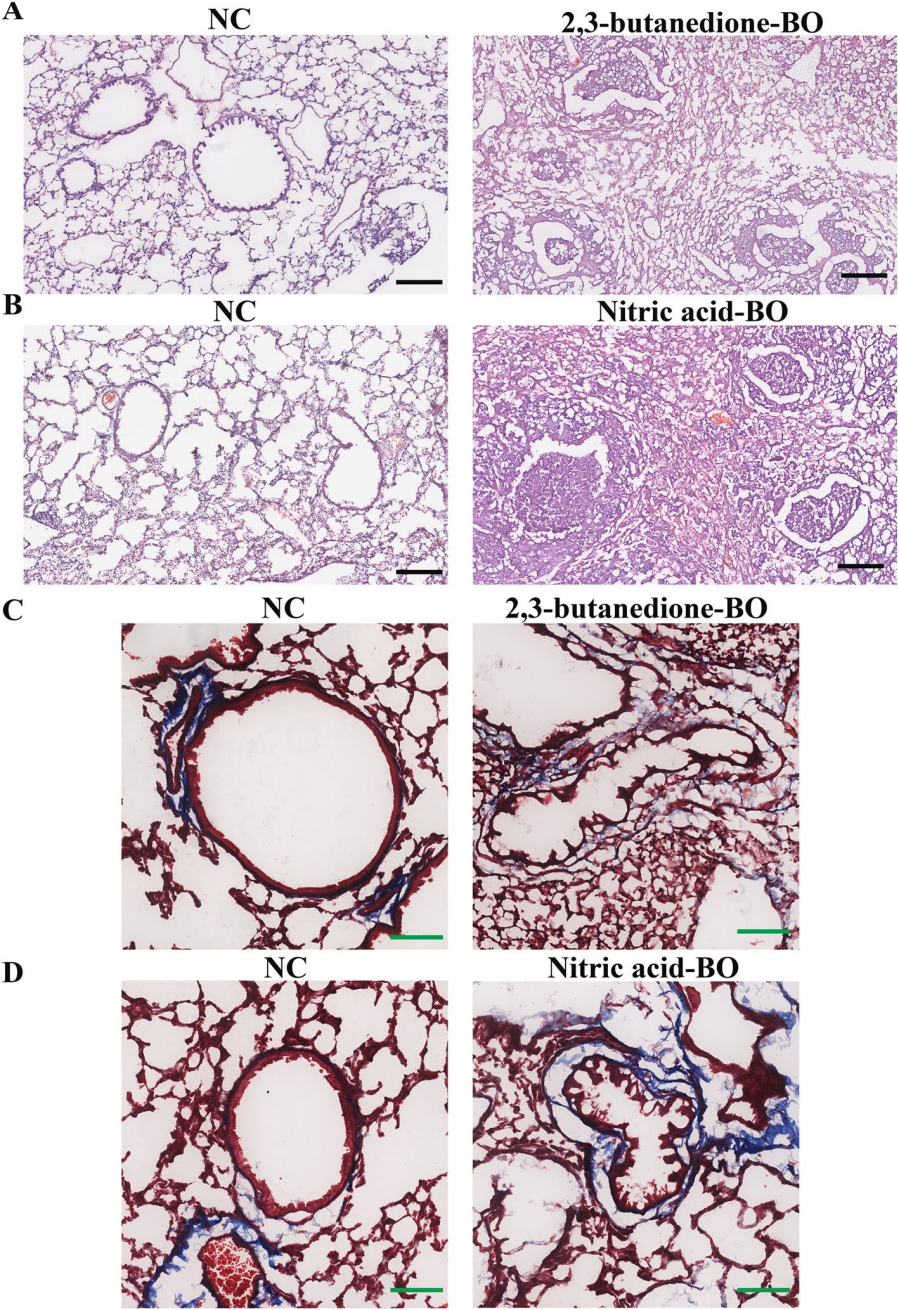
Pathological changes in the lungs from BO groups and NC groups. (**A**,**B**) HE staining shows obvious obstruction in small airways, with significant infiltration of inflammatory cells in the surrounding and luminal areas of affected small airways. (**C**,**D**) Mason’s trichrome staining shows more obvious fibrotic changes in the bronchi, with blue staining around the trachea indicating increased deposition of collagen protein and obvious narrowing and deformation of the tube wall. Tissues for A and C were obtained from 2, 3-butanedione-induced BO mice and their control group. Tissues for (**B**) and (**D**) were obtained from nitric acid-induced BO mice and their control group. In the HE-stained images, the scale bar represents a unit of 200 μm, while in the Masson-stained images, the scale bar represents a unit of 100 μm.

**Figure 6:**
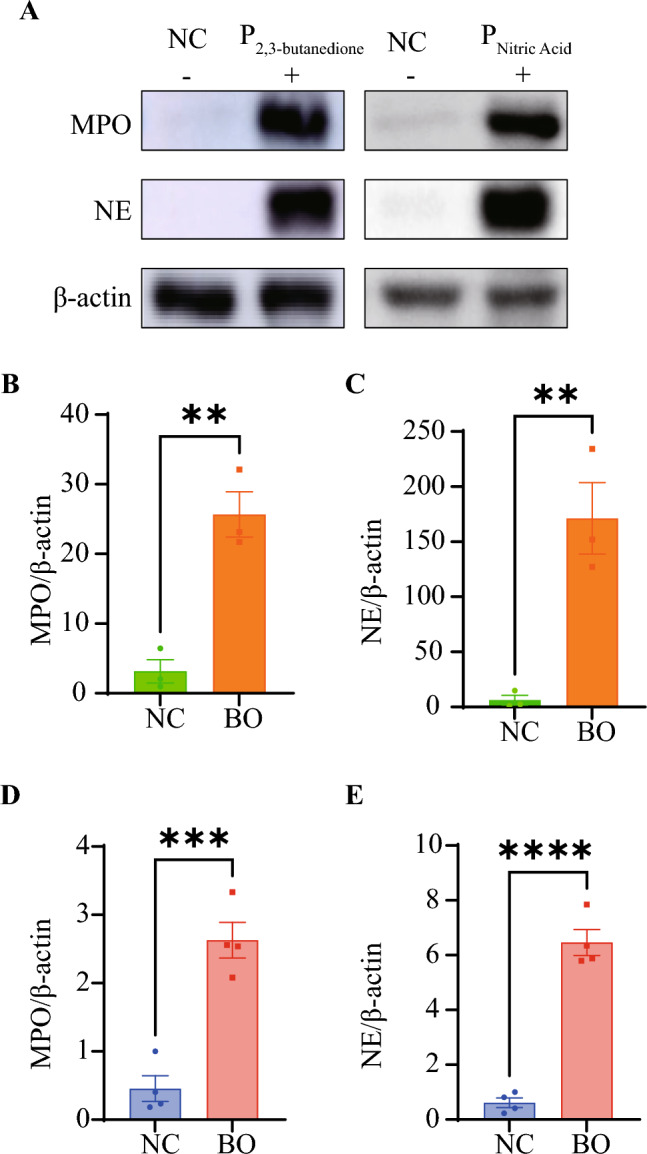
Levels of MPO and NE protein expression in the lung tissue of BO mice. (**A**) Representative western blot images. (**B**) Quantitative analysis of MPO western blot assay between 2, 3-butanedione-induced BO mice and their control group. (**C**) Quantitative analysis of NE between 2, 3-butanedione-induced BO mice and their control group. (**D**) Quantitative analysis of MPO western blot assay between nitric acid-induced BO mice and their control group. (**E**) Quantitative analysis of NE western blot assay between nitric acid-induced BO mice and their control group. Data shown mean ± SEM. n = 3–4/group*.* **p < 0.01, ***p < 0.001, ****p < 0.0001.

We successfully constructed mouse models of BO by administering 0.5% nitric acid and 30% 2,3-butanedione tracheal drips to mice, respectively. Representative photomicrographs of H&E stained sections of mouse lung demonstrating pathology following 2,3-butanedione exposure (n = 3) and nitric acid exposure (n = 4) (Fig. 5A,B); Representative photomicrographs of Masson’s stained sections of mouse lung demonstrating pathology following 2,3-butanedione exposure (n = 3) and nitric acid exposure (n = 4) (Fig. 5C,D). In both the BO model mice, we observed that more than 50% of the area of the diseased fine bronchioles was occluded (Supplementary Fig. 2A and B).These pathological changes indicate the development of BO in the mice, which could be used as a model for studying the mechanisms underlying the disease. The level of MPO and NE expression in the lung tissue of both 2,3-butanedione and nitric acid-induced BO mice was found to be significantly elevated (p < 0.01 and p < 0.001, respectively, for MPO; p < 0.01 and p < 0.0001, respectively, for NE) (Fig. 6B–E)compared to control mice. This has been illustrated with representative western blot images in Fig. 6A.

### DEGs were identified between small airway epithelial cells treated with NETs and untreated small airway epithelial cells

**Figure 7:**
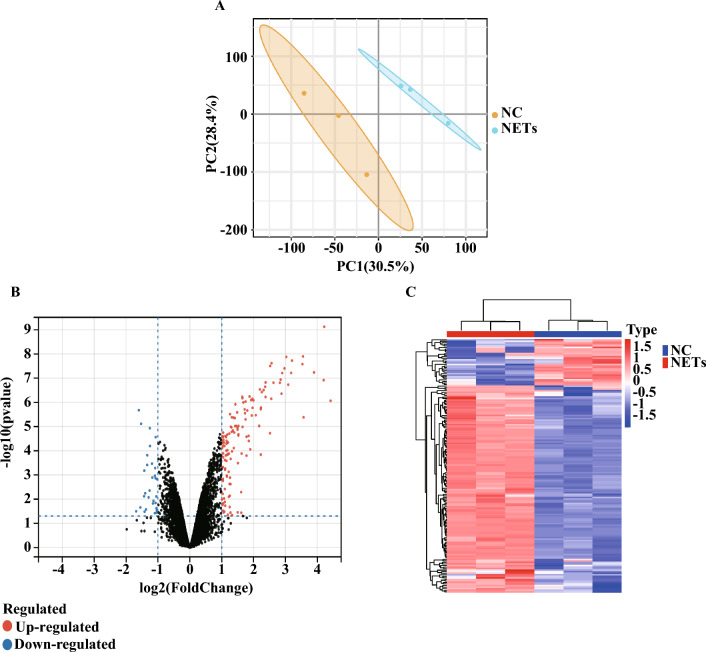
PCA in all SACEs and identification of NETs-DEGs between the untreated-SAECs and NETs-SAECs. (**A**) The volcano plots (**B**) and heat mapping (**C**) showed the DEGs between the untreat-SAECs and NETs-SAECs. n = 3/group.

The small airway epithelial cells treated with NETs (NETs-SAECs) were separated from untreated SAECs the in the principal component analysis (PCA) and the clustering heat mapping (Fig. 7A,C). Expression difference analysis revealed 134 up-regulated genes and 30 down-regulated genes in the NETs treatment group compared to the control group (Fig. 7B). The distribution of logFC and P values for the 164 NETs-DEGs was shown in the volcano and heat mapping.

### KEGG and GO results of NETs-DEGs

**Figure 8:**
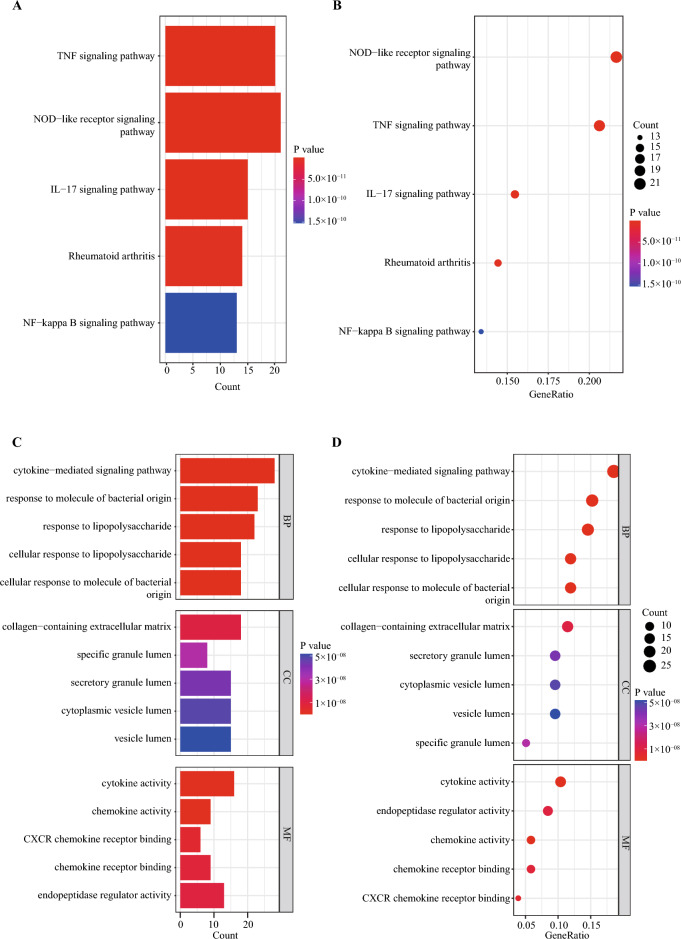
KEGG and GO enrichment results of NETs-DEGs. (**A**,**B**) The bar graph (**A**) and bubble plot (**B**) showed the top 5 significant pathways based on the P values in the KEGG analysis. (**C**,**D**) The bar graph (**C**) and bubble plot (**D**) showed the top 5 significant items in the BP, CC, and MF fractions based on the P values in the GO analysis.

We subjected the 164 NETs-DEGs to KEGG and GO analyses. KEGG pathway analysis revealed that the pathways enriched by NETs-DEGs include ‘TNF signaling pathway,’ ‘NOD-like receptor signaling pathway,’ ‘IL-17 signaling pathway,’ ‘Rheumatoid arthritis,’ and ‘NF-kappa B signaling pathway’ (Fig. 8A,B). According to the results of GO analysis results, the changes in BP of NETs-DEGs were significantly enriched in the ‘cytokine-mediated signaling pathway’, ‘response to molecule of bacterial origin’, ‘response to lipopolysaccharide’, ‘cellular response to lipopolysaccharide’ and ‘cellular response to molecule of bacterial origin’; the changes in CC of NETs-DEGs were enriched in ‘collagen-containing extracellular matrix’, ‘specific granule lumen’, ‘secretory granule lumen’, ‘cytoplasmic vesicle lumen’ and ‘vesicle lumen’, and the changes in MF were enriched in ‘cytokine activity’, ‘chemokine activity’, ‘CXCR chemokine receptor binding’, ‘chemokine receptor binding’ and ‘endopeptidase regulator activity’ (Fig. 8C,D).

### Protein–protein interaction network analysis of NETs-DEGs

**Figure 9:**
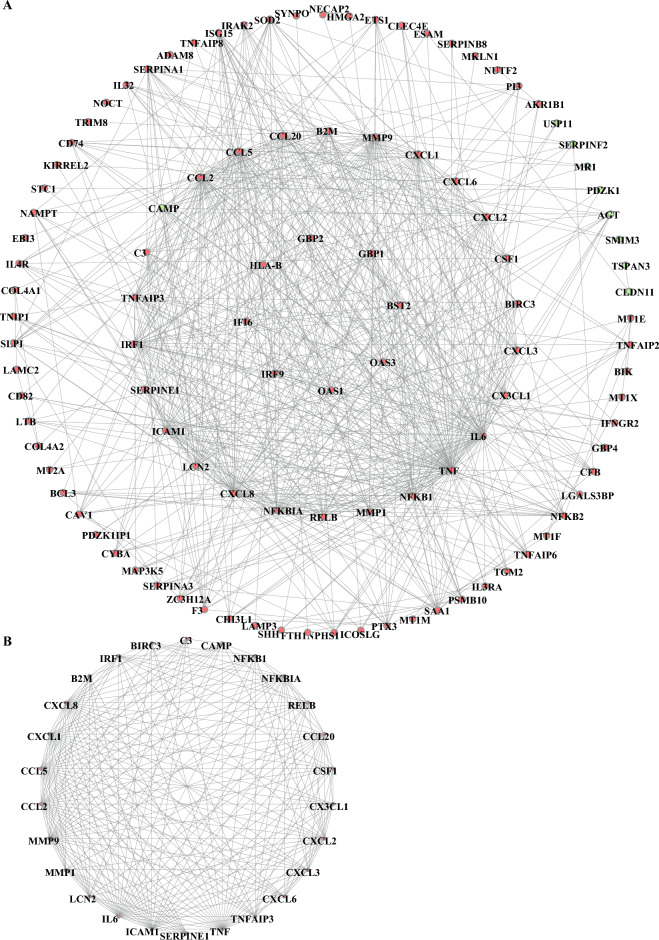
PPI network and function module identification. (**A**) PPI network displayed 107 NETs-DEGs in the PPI network after hiding disconnected nodes in the network, (**B**) One key module was identified based on MCODE analysis (MCODE score = 18), including 26 key NETs-DEGs.

We uploaded NETs-DEGs to the STRING online database to form the PPI network. We used the Cytoscape software to generate a PPI network. we built a PPI network with 107 NETs-DEGs after hiding disconnected nodes (Fig. 9A). We identified one key module based on MCODE analysis (MCODE score = 18), including 26 key NETs-DEGs: LCN2, MMP1, CX3CL1, ICAM1, CXCL2, TNFAIP3, IL6, CSF1, MMP9, CXCL8, C3, BIRC3, NFKB1, CXCL6, IRF1, NFKBIA, B2M, SERPINE1, CAMP, CCL20, CCL5, RELB, CCL2, TNF, CXCL1, and CXCL3. The remaining key NETs-DEGs are up-regulated, except for CAMP (Fig. 9B).

### KEGG analysis results of key module genes

**Figure 10:**
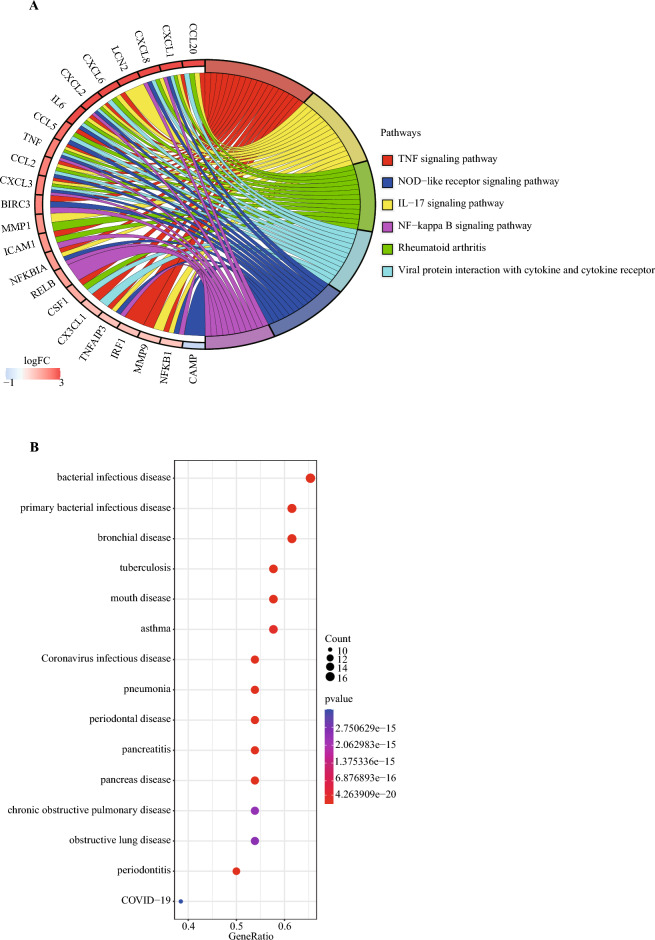
KEGG and DO enrichment results of key module genes. (**A**) Enrichment circle graph showed top 6 significant pathways based on the P values in the KEGG analysis of the key module genes. (**B**) The bubble plot showed top 15 significant diseases based on the P values in the DO analysis of the key module genes.

KEGG pathway analysis revealed that the top6 KEGG pathways enriched by key module genes include ‘TNF signaling pathway’, ‘IL-17 signaling pathway’, ‘Rheumatoid arthritis’, ‘Viral protein interaction with cytokine and cytokine receptor’, ‘NOD-like receptor signaling pathway’ and ‘NF-kappa B signaling pathway’ (Fig. 10A).

### Disease ontology analysis results of key module genes

DO analysis revealed that the disease enriched by key module genes include ‘bacterial infectious disease’, ‘primary bacterial infectious disease’, ‘bronchial disease’, ‘tuberculosis’, ‘mouth disease’, ‘asthma’, ‘Coronavirus infectious disease’, ‘pneumonia’, ‘periodontal disease’, ‘pancreatitis’, ‘pancreas disease’, ‘chronic obstructive pulmonary disease’, ‘obstructive lung disease’, ‘periodontitis’ and ‘COVID − 19’ (Fig. 10B).

## Discussion

BO is a rare chronic respiratory disease that can result in respiratory insufficiency^1^. Limited understanding of the basic underlying mechanisms modulated through BO pathogenesis had restricted current effective treatment options for BO patients. In order to gain further insights into BO pathogenesis, our study utilized combinatorial analytical approaches such as transcriptome sequencing and bioinformatics analysis to screen and explore differential gene expression changes and their respective underlying molecular mechanisms that were associated with the disease onset and progression. Our findings will provide novel therapeutic avenues into understanding the complex BO pathophysiology, ultimately contributing to the development of new therapeutic strategies for treating BO.

Our study managed to identify 115 up-regulated DEGs that are distinctive for BO pathogenesis, while additional GO analysis revealed that they were found involved in multiple inflammatory-related biological processes, such as leukocyte migration, neutrophil chemotaxis, regulation of tumor necrosis factor production, chemokine signaling pathway, and leukocyte transendothelial migration. The findings inevitably had provided the basis for further research into BO-associated inflammation regulatory signaling pathways. It is undeniable that inflammation plays pivotal roles for BO development and progression^1^. Pathological changes that were first observed in BO initiation are the infiltration of white blood cells such as lymphocytes, neutrophils, and eosinophils, into the peripheral airways, bronchial walls, and bronchioles^18^. Studies have revealed that the elevated sputum production in children with BO had a significantly increased neutrophil count with enhanced neutrophil chemokines levels such as IL-1β, IL-6, and IL-8^19^. Besides, clinical studies also found that the percentage of neutrophils and IL-8 in bronchoalveolar lavage fluid were significantly increased in post-measles BO patients, at the levels that were similarly observed in lung transplanted BO patients^20, 21^. Bioinformatics analysis from our study further reinforces the importance of inflammation regulatory networks, and at the same time, pointed out the the dominant roles of neutrophils in BO pathogenesis. By inhibiting neutrophils activation and subsequent inflammation activation, novel treatments to achieve complete recovery for BO patients may be possible.

Not surprisingly, our study found that NETs were activated in BO based on GSEA and KEGG analysis results. Briefly, the components of NETs are DNA, histones, NE, MPO, and various types of granule proteins^22^. While NETs help to trap and eliminate microbes in the lungs, persistent activation of NETs could lead to adverse health effects^23^. A study by Keir et al. had shown that the excessive formation and accumulation of NETs in lung tissue can lead to serious tissue damage due to constant inflammatory processes, which could promote the onset and progression of lung diseases^24^. Interestingly, no correlations have been established between NETs activation and BO pathogenesis. Our results further showed that core components of NETs, such as NE and MPO, were significantly up-regulated in the lungs of BO mice compared to healthy controls, suggesting that the NETs formation pathway may be involved in BO pathogenesis. Therefore, further studies are warranted to explore and dissect the specific role of NETs and their respective molecular mechanisms in BO. Completion of this study may provide the basis for new insights into the diagnosis and treatment of BO.

In our study, we also investigated the role of NETs in BO via the use of small airway epithelial cells, in order to confirm roles of NETs in BO by conducting transcriptome sequencing. Results from the transcriptome sequencing data had suggested that NETs may dysregulate multiple signaling pathways, including the TNF signaling pathway, NOD-like receptor signaling pathway, IL-17 signaling pathway, NF-kappaB signaling pathway, and the responses to molecule of bacterial origin. Consistent with Hudock’s study, we found that NETs could induce and promote TNF-α overexpression in SAEC^25^. Tumor necrosis factor, better known as TNF-α, is an important cytokine that was found involved in a series of inflammatory networks, and its activation can promote downstream activation of several biological effects that include the chemotaxis of inflammatory cells, stimulation of inflammatory factor production, oxidative and nitrosative stress response, and epithelial-mesenchymal transition^26–28^. Some studies had also demonstrated that TNF is elevated in sputum, blood, and bronchoalveolar lavage fluid from BO patients^19, 29^. Although considerable effort had been put into TNF, no substantial association with the occurrance and BO progression currently were found.

Besides, the NOD-like receptor (NLR) signaling pathway also plays an important role in the innate immune response against pathogens. A study of BO showed that the inhibition of the NLRP3 inflammasome can reduce airway inflammation and fibrosis^30^. Additionally, Park et al. also found that NLRP3 release by human bronchial epithelial cells can induce inflammation and EMT in asthma patients^31^. The research further highlighted the importance to understand the correlations between the NLR signaling pathway and pulmonary diseases. In addition, previous studies also had shown that NETs can activate macrophages and non-small cell lung cancer cells through the NLR pathway, leading to inflammation and EMT^32, 33^. However, the exact mechanisms of how NETs regulate the NLR signaling pathway in small airway epithelial cells were still lacking. Based on the observations from our results and the aforementioned studies, we hypothesize that NETs may activate the NLR signaling pathway in small airway epithelial cells, leading to inflammation and EMT activation.

Aside from that, a study from Wan et al. had demonstrated that NETs can stimulate IL-1, IL-2, and IL-8 overexpression in airway epithelial cells by activating the TLR4/NF-κB pathway^34^, which eventually leads to increased neutrophils recruitment and exacerbated inflammation. Accumulated studies have also revealed that IL-17 might be a part of NETs-associated molecules^35, 36^, in which the activation of bronchial epithelial cells by IL-17 activation had been reported to promote IL-1 and IL-8 releases, and consequently inducing neutrophilic inflammation^37^. In line with these findings, our results also demonstrated that NETs-treated airway epithelial cells can exhibit significant IL-1, IL-2, and IL-8 up-regulation, which may be attributed to NF-κB pathway and IL-17 signaling activation.

It is interesting to note that patients with rheumatoid arthritis (RA) history may also develop BO^38, 39^. Specifically, previous studies have demonstrated NETs were found to be enriched in the peripheral blood and synovial fluid of RA patients, in which the NETs overexpression significantly enhances the inflammatory reaction of RA synovial fibroblasts by inducing the production of IL-6, IL-8, chemokines, and adhesion molecules that leads to substantial joint damages^40, 41^. However, due to the rarity of RA-associated BO cases, the research to dissect the relationship between RA, BO, and NETs are substantially lacking. Hence, further experimental validations are needed to investigate the relationship between these diseases. We speculate that NETs may be a common mechanism that was activated, linking between RA and BO, in which RA induces BO occurrence by inducing NETs overexpressions in the lungs. Our study also provides a basis to explore the molecular mechanisms and target therapy of BO in RA patients.

Apart from chemokine activation and medical background, our study also revealed that NETs may amplify the response of airway epithelial cells to bacterial-derived molecules, such as lipopolysaccharide (LPS). LPS is a type of endotoxin-like biomolecule that exists in the outer membrane of many bacteria, which had been found to induce plethora of immune and inflammatory reactions^42, 43^. The production of various cytokines, such as TNF, IL-1, IL-6, and other inflammatory factors stemmed from the interaction between LPS and host cells may inevitably leads to prolonged inflammation and cell damage^44, 45^. Hence, we infer that NETs not only function to eliminate LPS invasion, but may also play a role in protecting the host from pathogenic bacterial invasion through activating and amplifying the response of human airway cells to LPS. However, considering that LPS is a strong inducer for NETs activation, there are instances that LPS may indirectly cause substantial damages to the airway due to the excessive NETs production by neutrophils upon stimulation. The idea to manipulate this cellular response into desired beneficial cell responses may, in turns, provide novel ideas to combat the mechanisms of infectious diseases involving NETs and LPS.

Lastly, our study had also constructed vital subnetworks of NETs associated DEGs, which were comprised of a set of 26 genes that were primarily involved in inflammatory regulatory networks and cellular chemotaxis factors. The pathways enriched by these genes were found to overlap with the pathways of NETs associated DEGs, indicating that they may play an essential role in the regulation of NETs associated DEGs. Furthermore, disease enrichment analysis also highlighted that these key subnetwork genes were highly associated with several airway or infectious diseases, including asthma, tuberculosis, pneumonia, coronavirus, bronchial diseases and chronic obstructive pulmonary disease, in which respective studies have shown a correlations between them and NETs^46–52^. This suggests that the negative impact of NETs on airway epithelial cells may be a common mechanism underlying the pathogenesis of these diseases including BO. However, additional research is still needed to fully understand the roles of NETs in these diseases and to identify potential therapeutic targets.

## Conclusions

In summary, our study had demonstrated significant correlation between NETs and BO, thereby suggesting a potential role for NETs in BO pathogenesis. Moreover, our findings also suggest that NETs may contribute to small airway epithelial cells dysregulation through the activation of inflammation and EMT related pathways. Although further investigations are required to validate and expand upon our observations, our report on the correlations between NETs and BO may provide novel insights into how the correlations may be exploited for future research and to develop better clinical management of BO.

### Supplementary Information

Supplementary Figure 1.

Supplementary Figure 2.

Supplementary Table 1.

Supplementary Table 2.

Supplementary Information.

## Data Availability

Publicly available datasets were analyzed in this study. These data can be found here: https://www.ncbi.nlm.nih.gov/geo/query/acc.cgi?acc=GSE52761. https://www.ncbi.nlm.nih.gov/geo/query/acc.cgi?acc=GSE137169. The raw RNA sequencing data of SAECs have been uploaded to the GEO database, and readers can access the dataset by entering a Reviewer token after the following website (https://www.ncbi.nlm.nih.gov/geo/query/acc.cgi?acc=GSE232805. Reviewer token: idgvasqibhwnzoz).
