# Supplementary material for: Transcriptome analysis reveals the impact of NETs activation on airway epithelial cell EMT and inflammation in bronchiolitis obliterans
Source: Sci Rep. 2023 Nov 6;13:19226. doi: 10.1038/s41598-023-45617-y (PMC10628238; doi:10.1038/s41598-023-45617-y)
Supplement: Supplementary file 1 — Supplementary Figure 1. [file 41598_2023_45617_MOESM1_ESM.pdf]

## Supplementary Figure 1

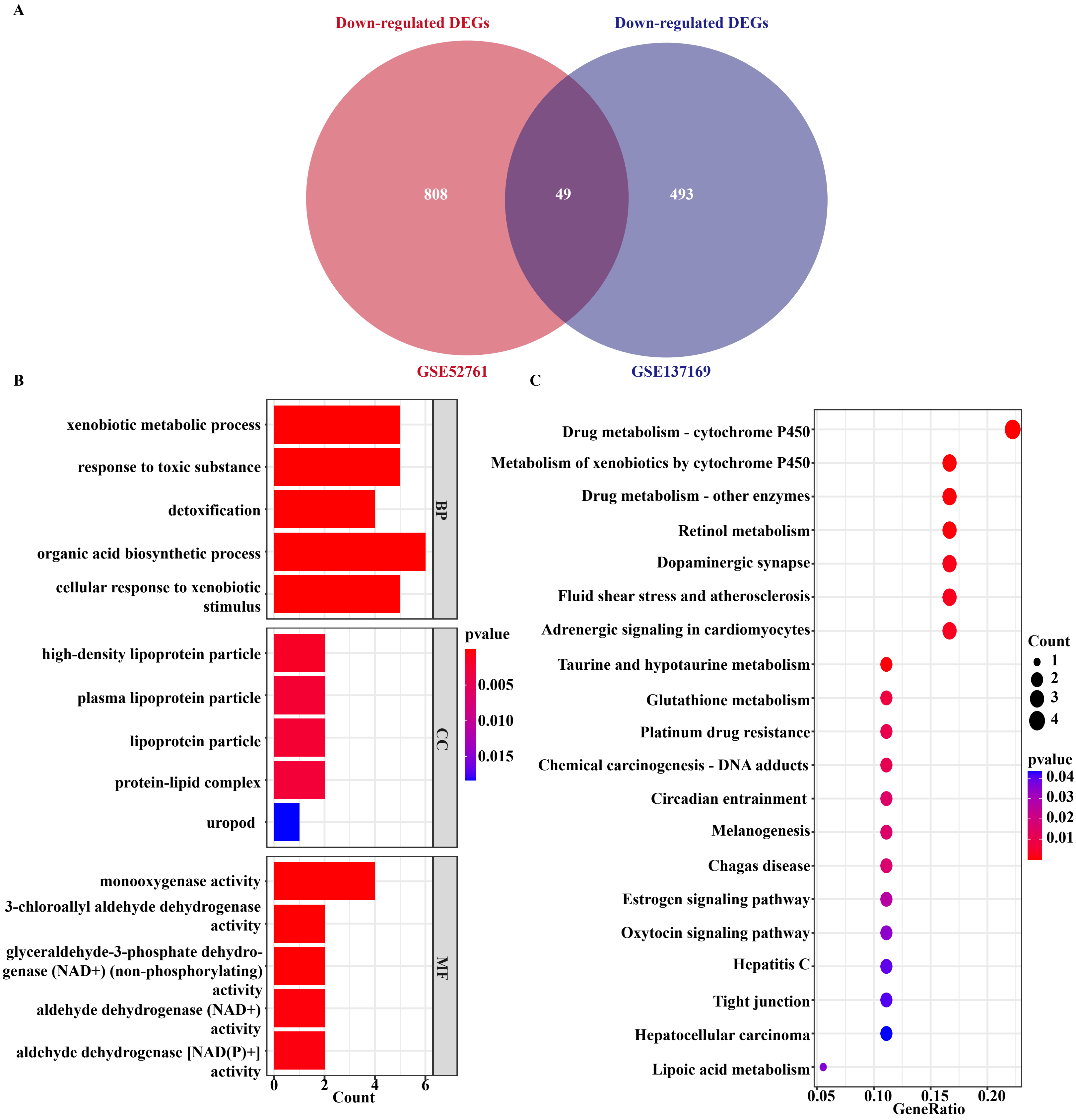


**Supplementary Figure 1**. Pathway Analysis of Down-Regulated Genes. A, Venn diagrams showed the overlap of numbers of down-regulated DEGs between GSE52761 and GSE137169. B, The bar graph showed the top 5 significant items in the BP, CC, and MF fractions based on the P values in the GO analysis for down-regulated DEGs. C, The bubble plot showed the top 20 significant pathways in the KEGG analysis for down-regulated DEGs.
