# Supplementary material for: Transcriptome analysis reveals the impact of NETs activation on airway epithelial cell EMT and inflammation in bronchiolitis obliterans
Source: Sci Rep. 2023 Nov 6;13:19226. doi: 10.1038/s41598-023-45617-y (PMC10628238; doi:10.1038/s41598-023-45617-y)
Supplement: Supplementary file 2 — Supplementary Figure 2. [file 41598_2023_45617_MOESM2_ESM.pdf]

Supplementary Figure 2

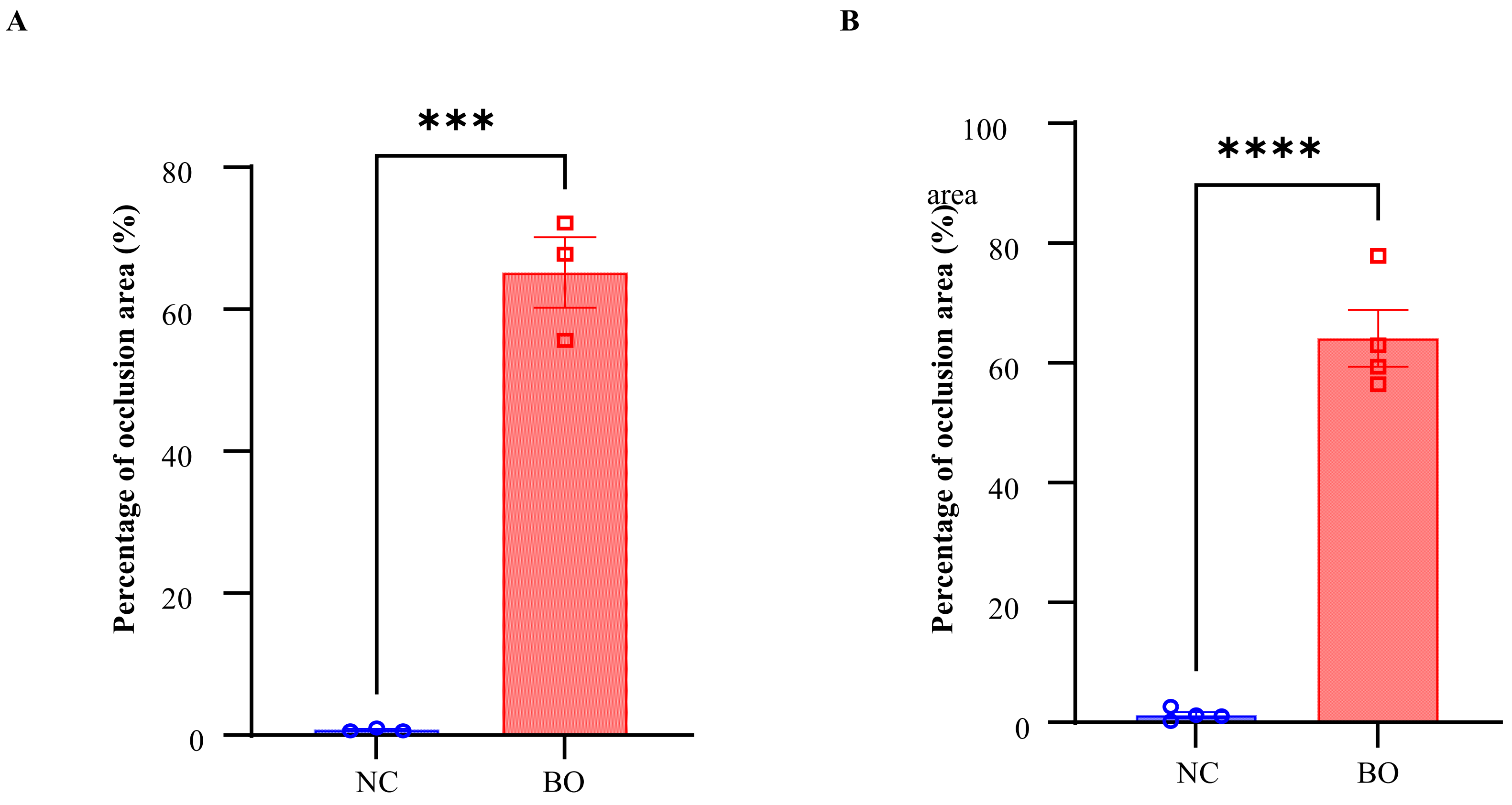

Supplementary Figure 2. Quantitative Analysis of Fine Bronchial Occlusion Area Percentage. A, Comparison of the degree of fine bronchial occlusion in 2,3-butanedione-induced BO mice and their control group. B, Comparison of the degree of fine bronchial occlusion in nitric acid-induced BO mice and their control group. ***$p < 0.001$, ****$p < 0.0001$.
