# Supplementary material for: Transcriptome analysis reveals the impact of NETs activation on airway epithelial cell EMT and inflammation in bronchiolitis obliterans
Source: Sci Rep. 2023 Nov 6;13:19226. doi: 10.1038/s41598-023-45617-y (PMC10628238; doi:10.1038/s41598-023-45617-y)
Supplement: Supplementary file 3 — Supplementary Table 1. [file 41598_2023_45617_MOESM3_ESM.doc]

**Supplementary Table 1**. Information Sheet on DEGs

| 115 Up-regulated DEGs | | | | | | | | | | | | |
| --- | --- | --- | --- | --- | --- | --- | --- | --- | --- | --- | --- | --- |
| Bcat1 | | Adgre1 | | Cst7 | | Fam167b | | Mki67 | | Melk | | Arhgap11a |
| Mafb | | Cx3cr1 | | Map4k1 | | Ctsz | | Ccnb1 | | Ccl17 | | Uhrf1 |
| Clec12a | | Itgb2 | | Nckap1l | | Ska1 | | Kif22 | | Slamf9 | | Cd68 |
| Tfec | | Cd33 | | Ccl2 | | C1qc | | Cenpk | | Arl11 | | Ciita |
| Trip13 | | Trem2 | | Ifi30 | | Igf1 | | Ccr5 | | Ccl22 | | Hcls1 |
| Clec4a3 | | Ccl7 | | Sh2b2 | | Gbp2 | | Espl1 | | Gpr84 | | Cybb |
| Ms4a7 | | Lum | | Tyrobp | | C3ar1 | | Strip2 | | Ccl6 | | Clec4a1 |
| Cdca3 | | Pld4 | | Nuf2 | | Prnd | | Arhgap30 | | Cdkn3 | | Apol9a |
| Cd300a | | Clec7a | | Kmo | | Apoe | | Clec9a | | Procr | | Fap |
| Mpeg1 | | Mmp12 | | Tlr8 | | Apobec1 | | Gcsam | | Rgs1 | | Tlr1 |
| Has2 | | Spc24 | | Mdk | | Gpnmb | | Pla2g2d | | Top2a | | Igsf6 |
| C1qb | | Cdca8 | | Cxcl13 | | Ctss | | Izumo1r | | Lilrb4 | | Birc5 |
| Epsti1 | | Ptafr | | Mmp7 | | Clec10a | | Tnfaip8l2 | | Cxcl10 | | Fcer1g |
| Shcbp1 | | Cdhr1 | | Thy1 | | Rab32 | | Kcnn4 | | Dtl | | Evi2a |
| C1qa | | Fcgr2b | | Tnfrsf9 | | Ptprv | | Mefv | | Bub1b | | Iqgap3 |
| Thbs2 | | Irf5 | | Slc26a4 | | Cpxm1 | | Pycard | | Fyb | | Vav1 |
| Cd83 | | Ccdc80 | | Ncf1 | |  | |  | |  | |  |
| 49 Down-regulated DEGs | | | | | | | | | | | | |
| Tspan13 | Icam2 | | Aldh1a1 | | Cyp4b1 | | Lgr6 | | Pllp | | Tmem212 | |
| Tspan12 | Fmo1 | | Cldn8 | | Ddah1 | | Pon1 | | Akap14 | | Zcchc12 | |
| Adgrl3 | Olfml2a | | Entpd3 | | Fgfbp1 | | Fmo3 | | Hp | | Cytl1 | |
| Azgp1 | Tppp3 | | Wif1 | | Ccdc81 | | Gstm7 | | Hs3st6 | | Calml3 | |
| Ces1e | Aldh1a7 | | Fam183b | | Gdpd2 | | Gmnc | | Cyp2s1 | | Trim54 | |
| Upk1a | Tnni3 | | Gnao1 | | Fbxo15 | | Stpg1 | | T2 | | Meig1 | |
| Ppp2r2b | Sec14l3 | | Prob1 | | Rbp4 | | Acsm1 | | Atp2c2 | | Gsta3 | |
