# Supplementary material for: Transcriptome analysis reveals the impact of NETs activation on airway epithelial cell EMT and inflammation in bronchiolitis obliterans
Source: Sci Rep. 2023 Nov 6;13:19226. doi: 10.1038/s41598-023-45617-y (PMC10628238; doi:10.1038/s41598-023-45617-y)
Supplement: Supplementary file 4 — Supplementary Table 2. [file 41598_2023_45617_MOESM4_ESM.docx]

**Supplementary Table 2**. Information Sheet on GSEA results

| 32 predicted BO up-regulated pathways from GSEA results | |
| --- | --- |
| Neutrophil extracellular trap formation | Rheumatoid arthritis |
| Primary immunodeficiency | Antigen processing and presentation |
| Graft-versus-host disease | Toll-like receptor signaling pathway |
| Hematopoietic cell lineage | Measles |
| Malaria | Leishmaniasis |
| Staphylococcus aureus infection | Phagosome |
| Allograft rejection | T cell receptor signaling pathway |
| Inflammatory bowel disease | p53 signaling pathway |
| Autoimmune thyroid disease | Fc gamma R-mediated phagocytosis |
| Viral protein interaction with cytokine and cytokine receptor | Chemokine signaling pathway |
| DNA replication | Complement and coagulation cascades |
| Intestinal immune network for IgA production | Th17 cell differentiation |
| Natural killer cell mediated cytotoxicity | Influenza A |
| Osteoclast differentiation | Cytosolic DNA-sensing pathway |
| B cell receptor signaling pathway | Pertussis |
| Systemic lupus erythematosus | Th1 and Th2 cell differentiation |
