# Supplementary material for: Transcriptome analysis reveals the impact of NETs activation on airway epithelial cell EMT and inflammation in bronchiolitis obliterans
Source: Sci Rep. 2023 Nov 6;13:19226. doi: 10.1038/s41598-023-45617-y (PMC10628238; doi:10.1038/s41598-023-45617-y)
Supplement: Supplementary file 5 — Supplementary Information. [file 41598_2023_45617_MOESM5_ESM.pdf]

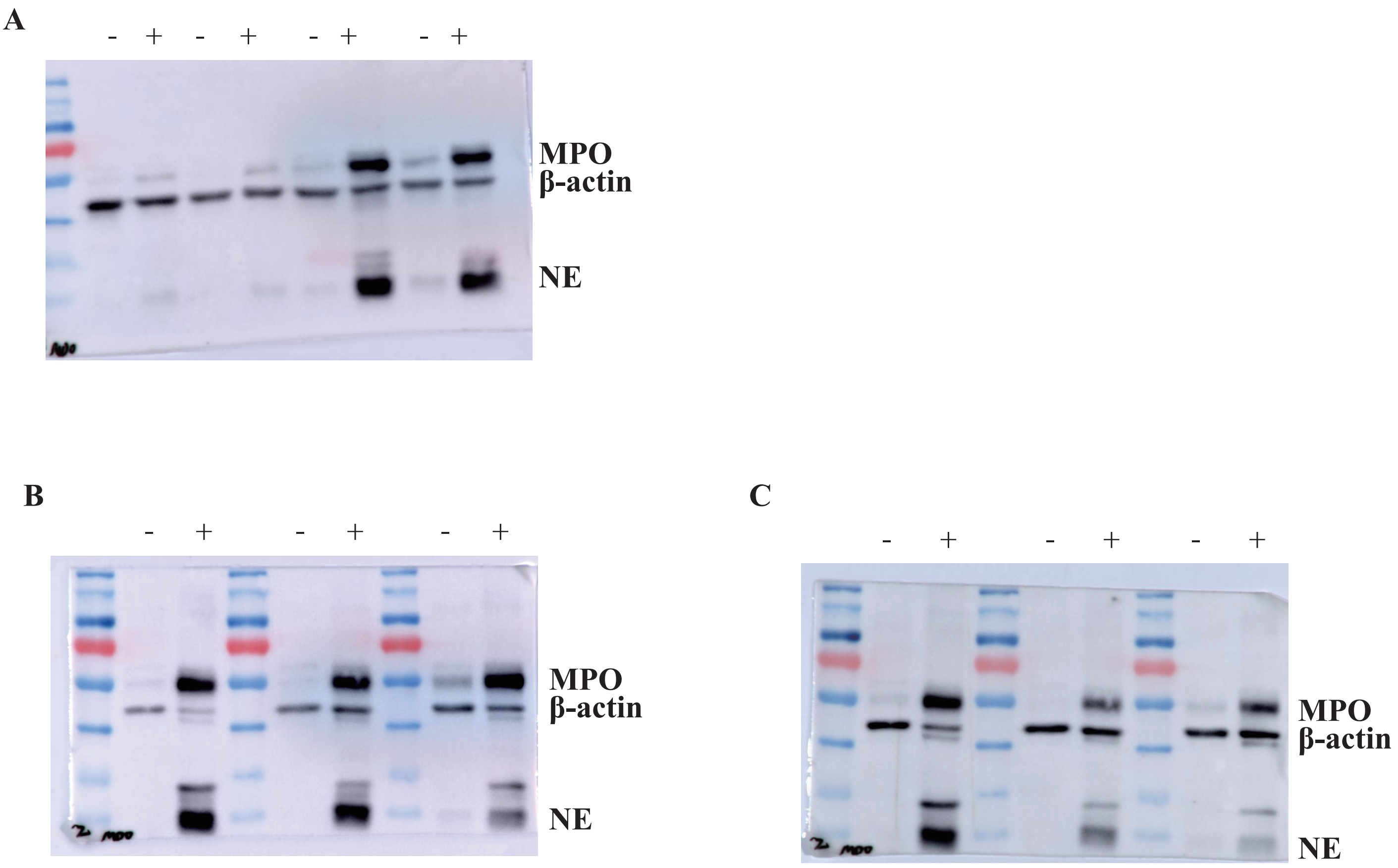

Unadjusted images of Westren Blot:

(A) shows the original Western Blot bands for the detection of NE (Neutrophil Elastase) and MPO (Myeloperoxidase) protein levels in lung tissues of BO mice induced with nitric acid. The Western Blot experiment included four control (NC) samples and four BO samples.

(B) shows the original Western Blot bands for the detection of NE (Neutrophil Elastase) and MPO (Myeloperoxidase) protein levels in lung tissues of BO mice induced with 2,3 butanedione. The Western Blot experiment included three control (NC) samples and three BO samples. (C) Due to the weak signal of β-actin in the first positive sample, we repeated the β-actin immunoblotting to enhance the signal.

Note: (1) A specific band was detected for MPO at approximately 67 kDa.
(2) A specific band was detected for NE at approximately 25 kDa.
(3) A specific band was detected for β-actin at approximately 42 kDa.
(4) The PageRuler pre-stained protein marker contains a range of proteins spanning from 10 to 180 kDa
